# The long non‐coding RNA LOC285758 promotes invasion of acute myeloid leukemia cells by down‐regulating miR‐204‐5p

**DOI:** 10.1002/2211-5463.12814

**Published:** 2020-03-26

**Authors:** Fangfang Xue, Haiyan Che

**Affiliations:** ^1^ Laboratory Medicine Jingmen No. 1 People’s Hospital China

**Keywords:** acute myeloid leukemia, invasion, lncRNA, LOC285758, miR‐204‐5p

## Abstract

Acute myeloid leukemia (AML) is the second most common type of leukemia worldwide. It was previously reported that expression of the long noncoding RNA LOC285758 is positively associated with AML cell proliferation, but the underlying mechanisms have not previously been reported. Here, we report that LOC285758 expression is higher in clinical AML blood samples and cultured AML cells. miR‐204‐5p was confirmed to be a target gene of LOC285758 by bioinformatics analysis and luciferase assay. LOC285758 overexpression promoted AML cell viability and invasion abilities, which were effectively inhibited by miR‐204‐5p overexpression; moreover, miR‐204‐5p overexpression also regulated the expression of E‐cadherin, N‐cadherin and Twist1. The data also showed that increased LOC285758 expression could effectively suppress the earlier effects of miR‐204‐5p on AML cells. Our findings suggest that targeting of miR‐204‐5p by LOC285758 promotes the cell viability and invasion of AML cells, and thus LOC285758 may have potential as a therapeutic target for AML treatment.

## Graphical abstract

The long noncoding RNA LOC285758 promotes cell viability and invasion of acute myeloid leukemia cells by down‐regulating miR‐204‐5p in acute myeloid leukemia.

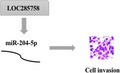

**Abbreviations**
AML: acute myeloid leukemia
CCK‐8: Cell Counting Kit‐8
DMEM: Dulbecco’s modified Eagle’s medium
lncRNA: long noncoding RNA
NC: negative control
q‐PCR: quantitative real‐time PCR
SD: standard deviation

Acute myeloid leukemia (AML) is the second most common type of leukemia worldwide 1, 2. The latest statistics revealed that the cure rate of AML in the young is 35%, whereas in the aged it is 15% by modern treatment strategies 3. AML, which is a highly heterogeneous hematopoietic malignancy with an annual incidence of approximately 3/100 000, remains a great challenge in clinical oncology 3, 4. The heterogeneity is also reflected in the lack of knowledge about detailed mechanisms of tumor formation and progression, resulting in less accurate classification and increased difficulty of deciding the appropriate treatment plan 5. In total, 60–80% of AML pathogenesis is associated with a chromosome translocation in somatic cells 1, 6. The dismal prognosis of AML motivates researchers to identify new genetic factors and investigate molecular mechanisms involved in AML pathogenesis, which may also contribute to the development of new therapeutic approaches and improvement of AML prognosis.

Long noncoding RNAs (lncRNAs) are a class of noncoding RNAs that share many features with protein‐coding RNAs (mRNAs) but lack open reading frame and are less conserved in their sequences and low expressed 5, 7. Evidence increasingly showed that abnormal expressions of lncRNAs are highly related to the occurrence and development of various diseases, indicating that lncRNAs can function not only as a typical oncogene, but also as tumor‐suppressive genes 8, 9. In addition, recent studies have proved that lncRNAs not only play a vital role in AML, but also served as biomarkers for early screening, diagnosis and treatment of AML 10, 11; for example, lncRNA HOXB‐AS3 was found to promote AML cell proliferation 12, lncRNA H22954 showed an inhibitory effect on AML cell growth via regulating Bcl‐2 family 13, and lncRNA PANDAR was correlated with a poor prognosis of AML 14. In addition, it was reported that lncRNA LOC285758 was high expressed in glioma cells in comparison with healthy brain cells and was negatively associated with promoter methylation in glioma 5, but positively associated with AML cell proliferation 1. However, the specific effects and mechanisms underlying LOC285758 in AML still need to be further investigated.

This study determined LOC285758 expression in AML and explored the effects and target gene of LOC285758 on AML cells. The purpose of this research was to determine the specific effects and mechanisms of LOC285758 in AML.

## Materials and methods

### Cell culture

Human bone marrow/stroma HS‐5 cell line and human AML cell lines (HL‐60, KG‐1a, NB4 and Kasumi‐1) were purchased from American Type Culture Collection (ATCC, Rockville, MD, USA) and cultured in Dulbecco’s modified Eagle’s medium (DMEM; C11995500BT; Gibco, Waltham, MA, USA) containing 10% FBS (10437010; Gibco) with 5% CO_2_ at 37 °C.

### Blood samples

Blood samples were gathered from the Department of Hematology of Jingmen No. 1 People’s Hospital between January 2017 and January 2019. All patients (*n* = 30) without other diseases were confirmed as having newly diagnosed AML (M0–M7) according to their pathological and clinical features, and the patients did not receive any antileukemic treatment before blood collection 10. The classification criteria for AML were based on standards developed by the French‐American‐British. The normal samples (*n* = 25) were obtained from healthy participants. Written informed consent was obtained from all research subjects, and the study was approved by the Ethics Committee of Jingmen No. 1 People’s Hospital. All procedures performed in studies involving human participants were in accordance with the ethical standards of the institutional and/or national research committee and with the 1964 Helsinki Declaration and its later amendments or comparable ethical standards.

### Transfection

lncRNA LOC285758 overexpression vector (LOC285758; lnc6180620115353‐1‐5), negative control (NC; lnc6N0000002‐1‐10), miR‐204‐5p mimics (Mimic; miR10022693‐1‐5) and Mimic control (miR01102‐1‐1) were purchased from RIBOBIO (Guangzhou, China). RNase‐free H_2_O (ST876; Beyotime, Shanghai, China) was used to dilute LOC285758, NC, Mimic and Mimic control to 20 μm and was stored at −20 °C for later use. Before the transfection, the cells (at 1.0 × 10^6^) were seeded into six‐well plates containing 2 mL complete medium to reach cell confluence of 60–70%. A total of 100 μL DMEM was used to dilute LOC285758 and Mimic to a concentration of 50 nm, whereas 3 μL Lipofectamine 2000 (11668‐019; Invitrogen, Carlsbad, CA, USA) was then added into another 100 μL DMEM. Then the two DMEMs were mixed together to a total volume of 200 μL and incubated for 15 min at normal temperature. Finally, the mixture was added into the cells of each well, which was then added with another 1.8 mL DMEM and further incubated for an additional 48 h. NC and Mimic control were conducted at the same time.

### RNA extraction and quantitative real‐time PCR

Total RNAs were extracted from the blood samples, HS‐5 and AML cells (HL‐60, KG‐1a, NB4 and Kasumi‐1) using TRIzol reagent (15596; Invitrogen) according to the reference instructions. Before the extraction, the blood samples were centrifuged (1 min, 10 000 ***g***) for collecting serum. Then serum and cells were lysed by TRIzol and transferred into a new 1.5‐mL centrifugal tube, and chloroform (C805334; Macklin, Shanghai, China) was then added into each tube and centrifuged at 14 000 ***g*** for 20 min. The supernatant was collected, mixed with an equal volume of isopropanol (H822173; Macklin) and centrifuged for 5 min (14 000 ***g***). RNA sediments were dissolved by RNase‐free H_2_O, and miRNAs were extracted from HS‐5 and AML cells (HL‐60 and KG‐1a) using a miRcute miRNA Isolation Kit (FP401; TianGEN, Beijing, China). In brief, the collected cells were seeded into a 1.5‐mL centrifugal tube and mixed with lysis buffer (provided by kit), and 200 μL chloroform was added into the tubes, which were then shaken for 1 min. After incubation for 5 min at normal temperature, the mixture was centrifuged for 20 min (13 400 ***g***), and the miRNA solution was collected into a new 1.5‐mL tube. Absolute ethanol (E801077; Macklin) was added into the tube, fully mixed with the solution and then centrifuged for 15 min (13 400 ***g***). The miRNA sediments were diluted using RNase‐free H_2_O.

PrimeScript RT kit (RR037A; Takara, Dalian, China) was used to reverse‐transcribe RNA into cDNA according to the instructions. Finally, gene expression was determined by quantitative real‐time PCR (q‐PCR) assays using Verso 1‐step RT‐qPCR Kit (A15300; Thermo Fisher Scientific, Inc., Carlsbad, CA, USA) in ABI 7500 Fast Real‐Time PCR System (Applied Biosystems, Foster City, CA, USA). The condition of q‐PCR was set at 95 °C for 30 s, followed by 60 °C for 30 s, 60 °C for 30 s for 45 cycles as described previously 10. GAPDH and U6 served as housekeeping genes for the analysis of lncRNA and miRNA expressions, respectively. All primer sequences are shown in Table 1. lncRNA and miRNA were quantified using the 2^−△△CT^ method 15.

**Table 1:** q‐PCR primers.

Target gene	Forward primers, 5′–3′	Reverse primers, 5′–3′
*LOC285758*	TTGTTTTTTGAAAGTTTTTTGA	AAACACAAAAAACCTAACAAAAA
*miR‐204‐5p*	CCACTTTGATTCCCTTTGTCATCC	CCACATTAGCGCGTATTCAGAC
*GAPDH*	GAAGGTGAAGGTCGGAGTC	GAAGATGGTGATGGATTTC
*U6*	CTCGCTTCGGCAGCACA	AACGCTTCACGAATTTGCGT

### Cell Counting Kit‐8 assays

Cell Counting Kit‐8 (CCK‐8; PA137267; Pierce, Rockford, IL, USA) was performed to determine cell viability. After the transfection, the cells (1.0 × 10^4^) were laid into 96‐well plates containing 100 μL complete medium. After cell growth for 24, 48 and 72 h, the cells were incubated by CCK‐8 reagent (0.5 mg·mL^−1^) for 15 min. Finally, the absorbance of each well was detected at 450 nm by a microplate reader (Infinite M200 PRO; Tecan Austria GmbH, Salzburg, Austria).

### Transwell assays

Transwell cell culture chambers were precoated with Matrigel (354234; Corning Life Sciences, Corning, NY, USA), and the inserts were placed into a 24‐well plate. The transfected cells were diluted to a density of 2 × 10^5^ cells and pipetted into the chambers that contained suspension solution with 0.2 mL FBS‐free Eagle’s Minimum Essential Medium, and the complete medium was added into the lower chamber. After incubation for 48 h, uninvaded cells at the upper side of the membrane were removed, leaving the underside of the membrane containing invaded cells. Finally, the invaded cells were stained by crystal violet for 15 min at normal temperature. The cells were counted from three random areas on each membrane at ×200 magnification under a phase‐contrast optical microscope (Axio Lab.A1 pol; Leica, Solms, Germany). image j software (Version 1.8.0; National Institutes of Health, Bethesda, MD, USA) was used to analyze the images.

### Western blot assays

Total proteins from the cells were isolated by radioimmunoprecipitation assay lysis buffer (P0013B; Beyotime), and the protein concentration was determined by bicinchoninic acid assay kit (23250; Pierce). Total proteins (30 µg) were separated on 10% SDS/PAGE gels, electroblotted and transferred to NC membranes (HTS112M; Millipore, Billerica, MA, USA). Then the membranes were blocked by 5% nonfat milk for 1 h at room temperature and incubated with antibodies [E‐cadherin (1 : 1000, ab40772, 97 kDa; Abcam, Cambridge, MA, USA), N‐cadherin (1 : 1000, ab18203, 130 kDa; Abcam), Twist1 (1 : 1000, ab50887, 21 kDa; Abcam) and GAPDH (1 : 1000, 36 kDa, ab8245; Abcam)] at 4 °C overnight. The next day, horseradish peroxidase–conjugated secondary antibodies [goat anti‐rabbit IgG secondary (1 : 5000, L3012; Signalway Antibody, College Park, MD, USA) and goat anti‐mouse IgG secondary (1 : 5000, ab205719; Abcam)] were incubated with the membranes for 1 h at room temperature. Finally, SuperSignal West Pico Chemiluminescent Substrate (Thermo Fisher Scientific, Inc.) was used to incubate the membranes for signal detection. image lab™ Software (version 3.0; Bio‐Rad Laboratories Inc., Hercules, CA, USA) was used for densitometric analysis and quantification of Western blot data (Bio‐Rad Laboratories Inc.).

### Statistical analysis

The data were processed by Dunnett’s *t*‐test and one‐way ANOVA in spss software (version 18.0; IBM, Armonk, NY, USA). The data were expressed as mean ± standard deviation (SD). All experiments were conducted in triplicate. A *P* value <0.05 was considered statistically significant.

## Results

### LOC285758 was high expressed in clinical blood samples derived from patients with AML and AML cell lines

**Fig. 1:**
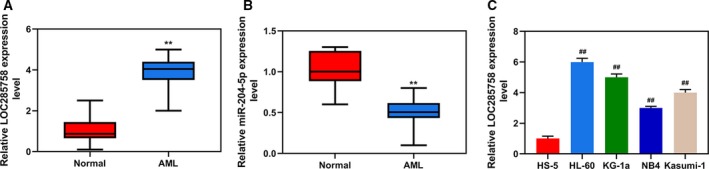
LOC285758 was high expressed in clinical blood samples derived from patients with AML and AML cell lines. (A) LOC285758 expression in AML and normal blood samples was analyzed by q‐PCR. (B) miR‐204‐5p expression in AML and normal blood samples was analyzed by q‐PCR. (C) LOC285758 expression in AML cells (HL‐60, KG‐1a, NB4 and Kasumi‐1) and normal bone marrow/stroma cells (HS‐5) was analyzed by q‐PCR. GAPDH served as a housekeeping gene of LOC285758. The experiments were conducted in triplicate; *n* = 3; SD was analyzed by Dunnett’s *t*‐test (**P < 0.01 versus normal; ^##^ *P* < 0.01 versus HS‐5).

LOC285758 and miR‐204‐5p expression levels in clinical AML blood samples and normal blood were compared by comparative analysis, and the results revealed that LOC285758 expression was obviously higher and miR‐204‐5p expression was lower in AML blood samples as compared with normal blood (*P* < 0.01; Fig. 1A,B). Consistently, as shown in Fig. 1C, the expression level of LOC285758 was higher in AML cells (HL‐60, KG‐1a, NB4 and Kasumi‐1) than that in normal bone marrow/stroma HS‐5 cells (*P* < 0.01). Among the AML cells, the expression of LOC285758 was the highest in HL‐60 and KG‐1a cells. Hence HL‐60 and KG‐1a cells were used in later experiments.

### LOC285758 overexpression promoted HL‐60 and KG‐1a cell viability and invasion

**Fig. 2:**
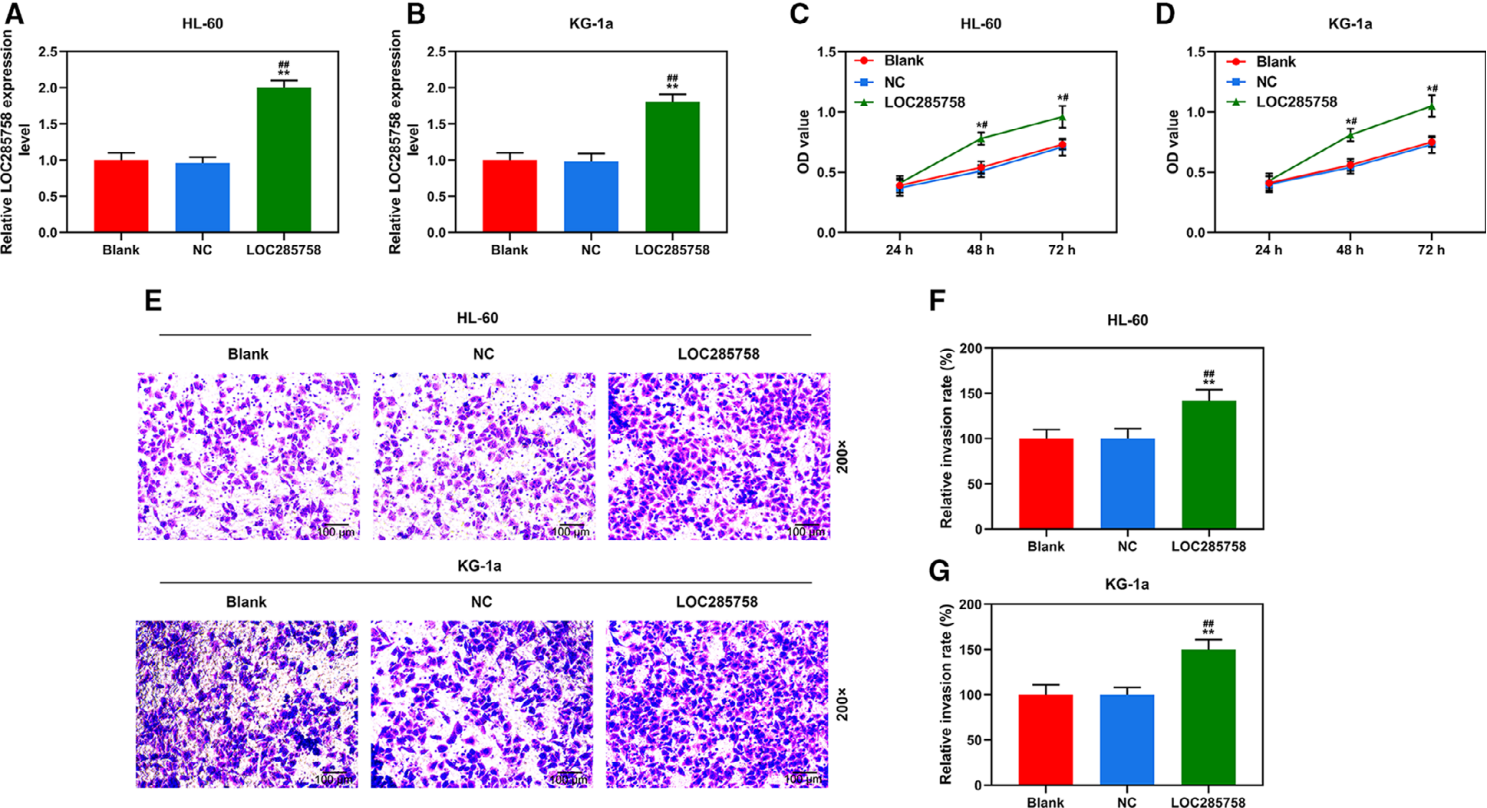
LOC285758 overexpression promoted HL‐60 and KG‐1a cell viability and invasion. (A, B) The transfection efficiency of LOC285758 in HL‐60 and KG‐1a cells was determined by q‐PCR; GAPDH served as a housekeeping gene of LOC285758. (C, D) Cell viabilities of HL‐60 and KG‐1a cells were measured using CCK‐8 assay after the transfection. (E–G) Cell invasion was analyzed by transwell assay in HL‐60 and KG‐1a cells after the transfection (original magnification ×200; scale bars: 100 μm). All experiments were conducted in triplicate; *n* = 3; SD was analyzed by Dunnett’s *t*‐test (*P < 0.05, **P < 0.01 versus blank; ^#^ *P* < 0.05, ^##^ *P* < 0.01 versus NC).

Because the miRNA‐204 was down‐regulated in AML patients, the mimics were applied in the research, so lncRNA was up‐regulated to reverse the changes brought by mimics. To examine the functions of LOC285758 in AML cells, we transfected HL‐60 and KG‐1a cells with LOC285758 overexpression vector (Fig. 2A,B); subsequently, the changes in cell viability and invasion were detected. As shown in Fig. 2C,D, we found that cell viabilities of blank and NC groups had no visible difference, whereas in the LOC285758 group, cell viabilities were significantly increased as compared with blank and NC groups (*P* < 0.05). As shown in Fig. 2E–G, we also found that the invasion of HL‐60 and KG‐1a cells was significantly promoted by LOC285758 overexpression compared with blank and NC groups (*P* < 0.01). The results suggested that LOC285758 overexpression could promote HL‐60 and KG‐1a cell viability and invasion.

### LOC285758 specifically targeted miR‐204‐5p

**Fig. 3:**
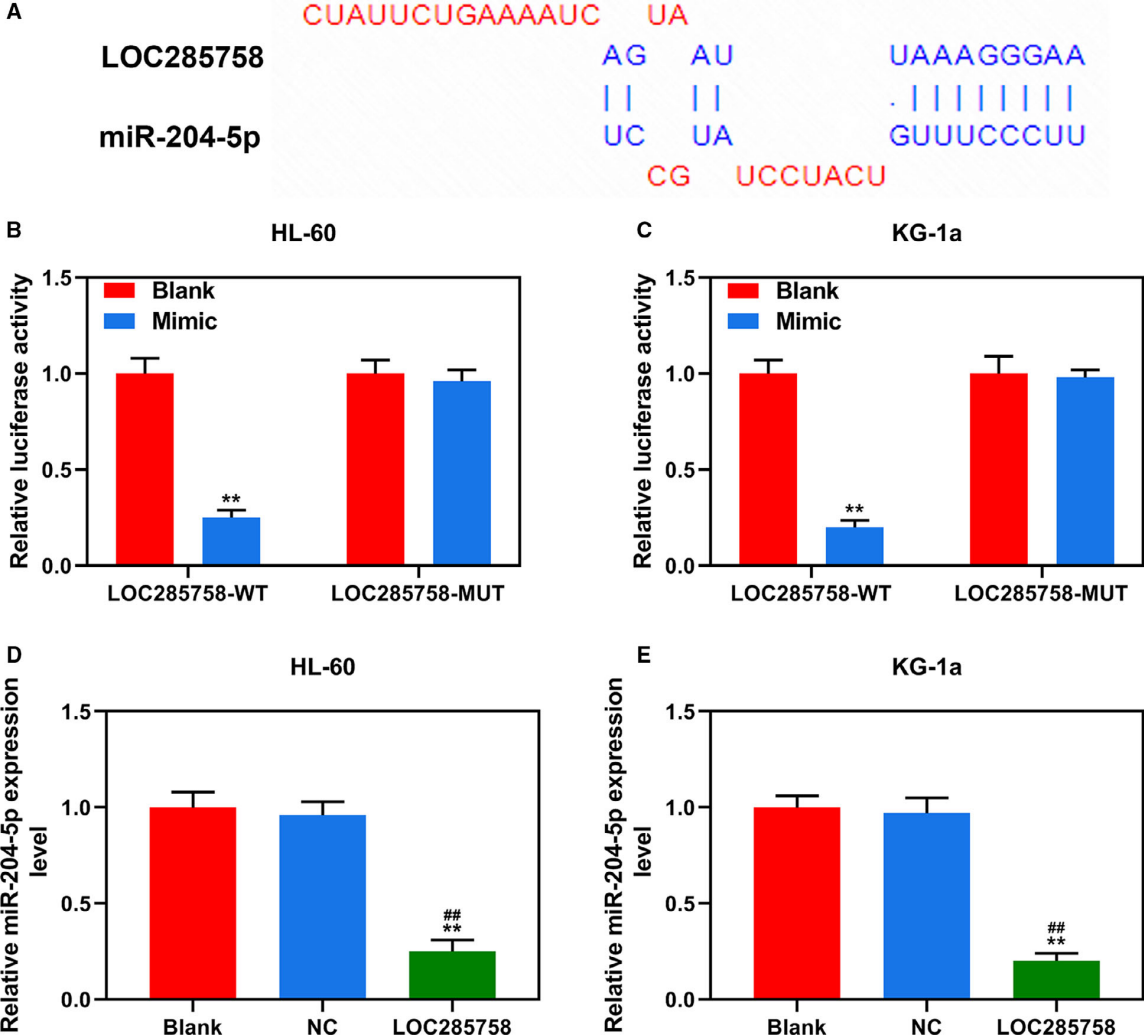
LOC285758 specifically targeted miR‐204‐5p. (A). miR‐204‐5p 3′ UTR contained binding sites of LOC285758. (B, C). Luciferase assay showed that LOC285758 targeted miR‐204‐5p in HL‐60 and KG‐1a cells; luciferase from firefly served as a reporter gene, and luciferase from sea kidney as an internal reference gene. (D, E) The expression of miR‐204‐5p in HL‐60 and KG‐1a cells was detected by q‐PCR assay after the transfection. U6 served as a housekeeping gene of miR‐204‐5p. All experiments were conducted in triplicate; *n* = 3; SD was analyzed by Dunnett’s *t*‐test (**P < 0.01 versus blank; ^##^ *P* < 0.01 versus NC). MUT, mutant; WT, wild type.

TargetScan predicted that miR‐204‐5p was a potential target of LOC285758, because it has a target sequence base pairing in miR‐204‐5p 3′ UTR (Fig. 3A). Luciferase reporter assays were conducted to further confirm the prediction; as shown in Fig. 3B,C, luciferase activity of HL‐60 and KG‐1a cells cotransfected with LOC285758‐wild type and miR‐204‐5p mimic were reduced compared with the blank group (*P* < 0.01). Moreover, after cotransfected with LOC285758‐mutant and miR‐204‐5p mimic, there was no difference in the luciferase activities between the Mimic and blank groups. The results indicated that miR‐204‐5p could be targeted by LOC285758. Subsequently, q‐PCR was further performed to detect the changes in miR‐204‐5p expression after overexpressing LOC285758. As shown in Fig. 3D,E, the expressions of miR‐204‐5p in HL‐60 and KG‐1a cells were remarkably down‐regulated in the LOC285758 group compared with the blank and NC groups (*P* < 0.01), confirming that LOC285758 specifically targets miR‐204‐5p.

### LOC285758 overexpression partly reversed the inhibitory effect of miR‐204‐5p on HL‐60 and KG‐1a cell viability and invasion

**Fig. 4:**
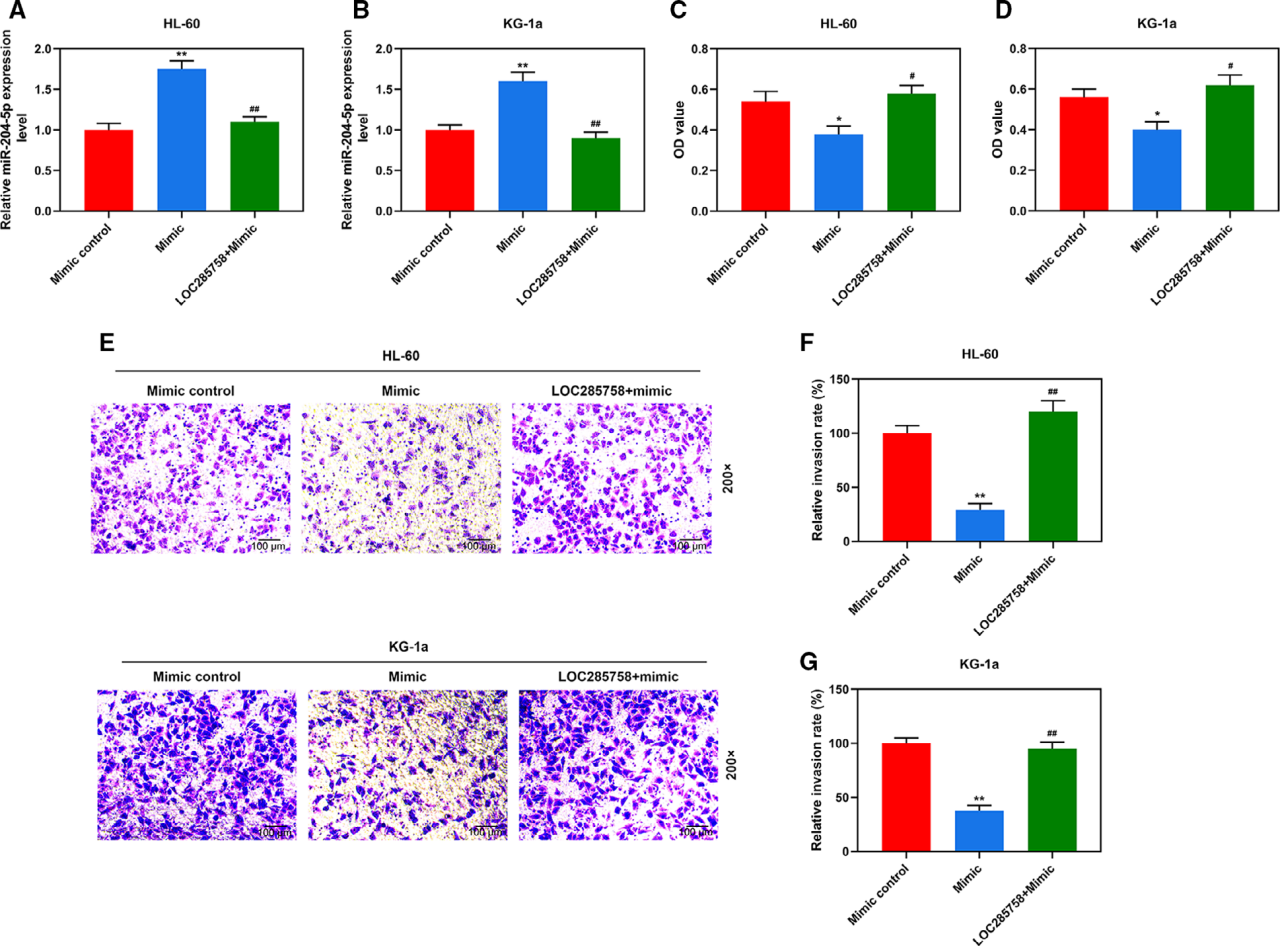
LOC285758 overexpression partly reversed the inhibitory effect of miR‐204‐5p on HL‐60 and KG‐1a cell viability and invasion. (A, B) The expression of miR‐204‐5p was detected by q‐PCR assay in HL‐60 and KG‐1a cells after the transfection. U6 served as a housekeeping gene of miR‐204‐5p. (C, D) Cell viability was determined by CCK‐8 assay in HL‐60 and KG‐1a cells after the transfection. (E–G) Cell invasion of HL‐60 and KG‐1a cells was analyzed by transwell assay after the transfection (original magnification ×200; scale bars: 100 μm). All experiments were conducted in triplicate; *n* = 3; SD was analyzed by Dunnett’s *t*‐test (*P < 0.05, **P < 0.01 versus mimic control; ^#^ *P* < 0.05, ^##^ *P* < 0.01 versus mimic).

CCK‐8, wound healing and transwell assays were performed to further evaluate whether the effects of LOC285758 on HL‐60 and KG‐1a cells were mediated through down‐regulating miR‐204‐5p. As shown in Fig. 4A,B, after transfection with miR‐204‐5p mimic, the expression levels of miR‐204‐5p in HL‐60 and KG‐1a cells were noticeably up‐regulated compared with mimic control (*P* < 0.01), indicating that miR‐204‐5p mimic was successfully transfected into the two cells. However, in the LOC285758 + mimic group, miR‐204‐5p expression was reduced in two cells by LOC285758 overexpression compared with the mimic group (*P* < 0.01), which further confirmed that LOC285758 could significantly down‐regulate the expression of miR‐204‐5p. In addition, Fig. 4C,D shows that cell viabilities of the two cells of the mimic group were greatly reduced compared with the Mimic control group (*P* < 0.05), whereas cell viabilities were significantly increased by LOC285758 overexpression in the LOC285758 + mimic group compared with the Mimic group (*P* < 0.05). Moreover, cell invasion of HL‐60 and KG‐1a cells was reduced in the Mimic group compared with the Mimic control group (*P* < 0.05), but remarkably increased by LOC285758 overexpression in the LOC285758 + Mimic group compared with the Mimic group (*P* < 0.01; Fig. 4E–G). Thus, the results showed that the promoting effects of LOC285758 on HL‐60 and KG‐1a cell viability and invasion were mediated through down‐regulating miR‐204‐5p.

### LOC285758 overexpression partly reversed the regulatory effect of miR‐204‐5p on the expressions of E‐cadherin, N‐cadherin and Twist1

**Fig. 5:**
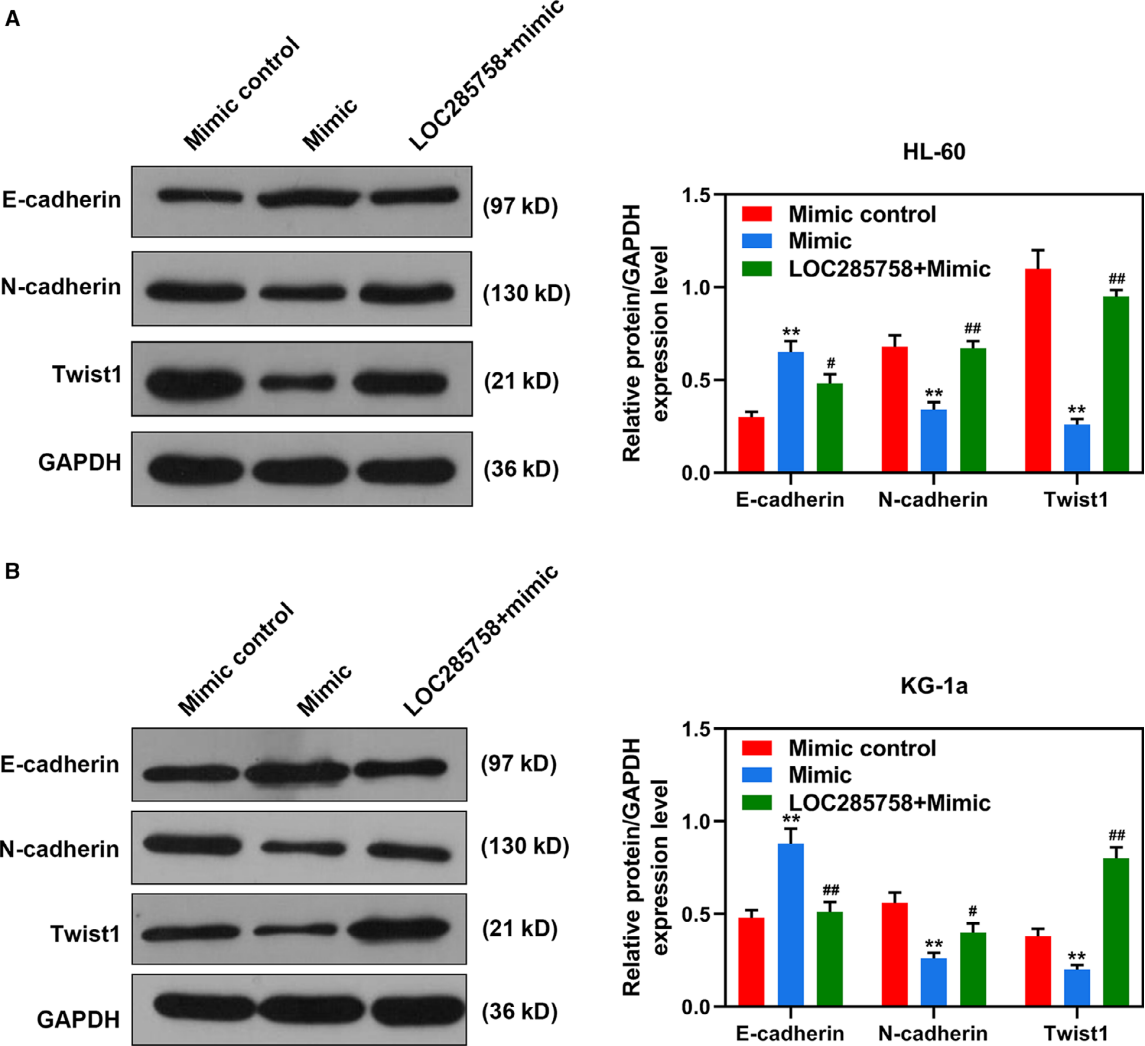
LOC285758 overexpression partly reversed the regulatory effect of miR‐204‐5p on the expressions of E‐cadherin, N‐cadherin and Twist1. The protein expressions of E‐cadherin, N‐cadherin and Twist1 in HL‐60 (A) and KG‐1a (B) cells were detected by Western blot assay after the transfection. GAPDH served as an internal control of proteins. All experiments were conducted in triplicate; *n* = 3; SD was analyzed by Dunnett’s *t*‐test (**P < 0.01 versus mimic control; ^#^ *P* < 0.05, ^##^ *P* < 0.01 versus mimic).

We further detected the expression levels of E‐cadherin, N‐cadherin and Twist1. As shown in Fig. 5A,B, in the Mimic group, the expression of E‐cadherin was up‐regulated, but the expressions of N‐cadherin and Twist1 were down‐regulated compared with the Mimic control group (*P* < 0.01). In the LOC285758 + Mimic group, the effects of miR‐204‐5p on these proteins of HL‐60 and KG‐1a cells were reversed by LOC285758 compared with the Mimic group (*P* < 0.05). The results proved that LOC285758 partly reversed the inhibitory effect of miR‐204‐5p on the expressions of E‐cadherin, N‐cadherin and Twist1, further confirming that the regulatory effects of LOC285758 on the cell invasion of HL‐60 and KG‐1a cells were associated with the inhibition of miR‐204‐5p.

## Discussion

Among various types of leukemia, many lncRNAs have been identified and studied in relation to several target protein‐coding genes in rearranged childhood acute lymphoblastic leukemia 16. In addition, in the progression of AML, MONC and MIR100HG were proved to be the regulators of erythro‐megakaryocytic development and contribute to the growth of leukemia 17. In acute promyelocytic leukemia, HOTAIRM1, an intergenic lncRNA, was proved to regulate cell proliferation 18. Some lncRNAs are involved in the pathogenesis of AML, for example, lncRNA CCDC26, which was proved to be overexpressed in childhood AML and have the ability to regulate c‐kit expression and control the growth of AML cells 19. Also, lncRNA LOC285758 expression has been confirmed to predict an overall prognosis for patients with AML 1. However, to the best of our knowledge, there is a lack of study about the specific effects and mechanism of LOC285758 in AML. Thus, this study focused on evaluating the effects and mechanism of LOC285758 in AML. We found that LOC285758 was high expressed in clinical AML blood samples and cultured AML cells, which was consistent with previous reports 1, 5. Subsequently, we explored the effects of LOC285758 overexpression on biological abilities of AML progression and found that LOC285758 overexpression remarkably increased the viability and invasion of AML cells, suggesting that the regulatory effects of LOC285758 on AML were mediated through affecting the malignant abilities of AML cells.

Increasing evidence showed that lncRNAs could competitively bind to certain miRNAs and subsequently regulate miRNA‐mediated downstream target gene silencing at the posttranscriptional level 20. miRNAs are small noncoding RNAs that have regulatory functions with 18–25 nucleotides in length 21. Since its discovery by Lee *et al.* 22, they have been reported to have important functions because they regulate 30–60% of all human gene expressions and are vital regulators in various biological functions of cells (e.g., cell proliferation, angiogenesis, metastasis and apoptosis) 21. Recently, researchers have proved the potential value of lncRNAs and miRNAs in cancer screening, diagnosis, and treatment; for example, lncRNA PVT1 was found to regulate the progression of pulpitis by down‐regulating the expression of miR‐455‐5p 23, and lncRNA DCRF could regulate cardiomyocyte autophagy through targeting miR‐551b‐5p 24. Bioinformatics algorithm found that miR‐204‐5p was a target gene of LOC285758. miR‐204 was previously reported to be involved in a series of physiological and pathological processes, such as diabetic retinopathy, aortic valve disease and ulcerative disease 25, 26, 27. Some recent studies revealed that miR‐204‐5p also exerts antitumor effects on various cancers (e.g., breast cancer, melanoma and liver cancer) through inhibiting invasion and metastasis of cancer cells 25, 28, 29, 30. This study found that miR‐204‐5p suppressed cell viability and invasion, and regulated the expressions of E‐cadherin, N‐cadherin and Twist1; moreover, LOC285758 could reduce the transcription of miR‐204‐5p and promoted AML cell viability and invasion.

## Conclusions

The current research reveals that lncRNA LOC285758 had high expression in clinical AML blood samples and cultured AML cells. LOC285758 overexpression not only promotes AML cell viability and invasion, but also notably down‐regulates the transcription of miR‐204‐5p, suggesting that LOC285758 promotes the progression of invasion of AML cells by competitively binding to miR‐204‐5p. Thus, LOC285758 might be a therapeutic target for AML treatment.

## Conflict of interest

The authors declare no conflict of interest.

## Author contributions

FX was involved in substantial contributions to conception and design, data acquisition, data analysis and interpretation. FX and HC drafted the article or critically revised the important intellectual content. All authors were involved in the final approval of the version to be published. HC was involved in the agreement to be accountable for all aspects of the work in ensuring that questions related to the accuracy or integrity of the work are appropriately investigated and resolved.

## Data Availability

The analyzed datasets generated during the study are available from the corresponding author on reasonable request.
